# Reduced intradisc vessel density is associated with optic disc hemorrhage in eyes with primary open-angle glaucoma

**DOI:** 10.1038/s41598-023-28288-7

**Published:** 2023-01-23

**Authors:** Jin Yeong Lee, Kyung Rim Sung, Joong Won Shin, Ko Eun Kim, Joon Mo Kim

**Affiliations:** 1 Department of Ophthalmology, HanGil Eye Hospital, Incheon, Korea; 2 Department of Ophthalmology, Asan Medical Center, University of Ulsan College of Medicine, 88 Olympic-ro 43-gil, Songpa-gu, Seoul, 05505 Korea; 3 Department of Ophthalmology, J′S Eye Clinic, Gangneung-si, Gangwon-do Korea; 4 Department of Ophthalmology, Kangbuk Samsung Hospital, Sungkyunkwan University School of Medicine, 29 Saemunan-ro, Jongno-gu, Seoul, 03181 Korea

**Keywords:** Diseases, Medical research

## Abstract

We sought to investigate the association between optic nerve head (ONH)/choroidal microvasculature perfusion and optic disc hemorrhage (ODH) in eyes with primary open-angle glaucoma (POAG) using swept-source optical coherence tomography angiography (SS-OCTA). A total of 266 POAG eyes (59 with a single instance of ODH, 40 with a history of recurrent ODH, and 167 eyes without ODH) with a mean follow-up of 5.4 years were included. Intradisc vessel density (VD), parapapillary choroidal VD, optic disc microvascular dropout (MvD), and choroidal microvascular dropout (CMvD), were evaluated on a 3 × 3 mm SS-OCTA image of ONH and compared between eyes with and without ODH. Recurrent ODH was defined as occurrence 1 year after first ODH detection during the total follow-up period. Logistic regression analyses were performed to investigate factors associated with ODH. The prevalence of CMvD, optic disc MvD, and β-parapapillary atrophy were not different among the no ODH, single ODH, and recurrent ODH groups. Eyes with ODH had lower intradisc VDs than those without ODH (*P* = 0.021), but no difference was found in intradisc VDs between the single and recurrent ODH groups (*P* = 0.977). Better VF MD at baseline (odds ratio [OR], 1.150; 95% confidence interval [CI], 1.055–1.254; *P* = 0.002) and lower intradisc VD (OR, 0.863; 95% CI, 0.812–0.918; *P* < 0.001) were associated with ODH occurrence. Among POAG eyes, those with ODH had lower intradisc VDs than those without ODH. POAG eyes in an earlier disease stage or those with lower intradisc VDs should be monitored for the possibility of ODH occurrence.

## Introduction

Primary open-angle glaucoma (POAG) is a chronic progressive optic neuropathy characterized by the loss of retinal ganglion cells and their axons, leading to visual field (VF) defects^1^. Although increased intraocular pressure (IOP) has been noted as the predominant risk factor for its development and progression, the potential role of reduced optic nerve head (ONH) perfusion has also been continuously investigated^2–4^. Recently, several studies using optical coherence tomography angiography (OCTA) have revealed the regional association between microvascular dropout (MvD) in the deep parapapillary layer, retinal nerve fiber layer (RNFL) defects, and decreased peripapillary vascular density (VD) in glaucomatous eyes, suggesting that microvascular changes may influence the glaucoma pathogenesis^5–7^.

Optic disc hemorrhage (ODH) is a well-known risk factor for glaucoma development and disease progression^8–10^. Recent studies have reported an association of ODH and vascular pathogenesis in glaucoma using OCTA. The prevalence of choroidal microvascular dropout (CMvD) was significantly higher in POAG eyes with ODH compared to POAG eyes without ODH^11,12^. Moreover, a topographic correlation has been found between CMvD and ODH location in glaucomatous ONH^11,12^. These findings suggest that both CMvD and ODH may share the vascular pathogenesis of glaucomatous damage. We hypothesized that ODH may be associated with the microvascular loss in ONH along with that in the parapapillary choroid represented as CMvD.

In the present study, we used swept-source (SS)-OCTA, which has the advantages of deeper penetration and higher resolution^13^ to assess microvascular perfusion in the ONH region of POAG eyes with and without ODH during follow-up, and we evaluated the association between ODH and microvascular changes within the optic disc.

## Methods

We conducted a retrospective review of electronic medical records of patients diagnosed with POAG who underwent SS-OCTA examination at the glaucoma clinic of Asan Medical Center from May 2020 to June 2021. The study protocol and the waiver of written informed consent were approved by the institutional Review Board of Asan Medical Center and this study adhered to the tenets of the Declaration of Helsinki.

### Participants

All participants underwent comprehensive ophthalmic examinations, including slit-lamp biomicroscopy, IOP measurement with Goldmann applanation tonometry, gonioscopy, refraction error measurement, central corneal thickness assessment with ultrasonic pachymetry (SP-3000; Tomey, Nagoya, Japan), dilated color fundus photography (Canon, Tokyo, Japan), ONH stereoscopic photography/red-free RNFL photography (Canon), and axial length measurement with the IOLMaster (Carl Zeiss Meditec Inc., Dublin, CA, USA), respectively. Patients were also examined using the Cirrus HD spectral-domain OCT system (Carl Zeiss Meditec Inc.), Humphrey field analyzer with Swedish Interactive Threshold Algorithm 24–2 VF testing (Carl Zeiss Meditec Inc.), and PLEX Elite 9000 version 1.7 SS-OCTA system (Carl Zeiss Meditec Inc.). IOP was measured at each visit, and the mean IOP was defined as the average of all IOP measurements during follow-up. Clinical history was collected from participants, including demographic characteristics and the presence of cold extremities, migraine, and other systemic conditions.

For inclusion in the current study, all patients had to meet the following criteria: (1) age > 18 years; (2) presence of open-angle glaucoma by gonioscopic examination; (3) glaucomatous optic nerve damage (i.e., neuroretinal rim thinning, notching, and/or RNFL defect); (4) glaucomatous VF defect defined by Anderson’s criteria (i.e., three or more adjacent points on a pattern deviation probability map with *P* < 0.05 and one abnormal point with *P* < 0.01, a Glaucoma Hemifield Test result outside the normal limits, or a pattern standard deviation of *P* < 0.05 confirmed on 2 consecutive reliable VF tests)^14^ in ≥ 2 reliable tests (fixation loss rate < 20% and false-positive and false-negative error rates < 15%) corresponding to glaucomatous structural damage; and (5) > 3 years of follow-up with regular OCT and VF exams at 6-month intervals.

Exclusion criteria were as follows: (1) eyes with spherical refraction < − 6.0 diopters (D) or ≥ + 3.0 D and cylinder correction < − 3.0 D or ≥ + 3.0 D; (2) eyes with other intraocular or neurologic diseases that affected RNFL thickness measurements; and (3) eyes with any history of intraocular surgery other than simple cataract surgery. If both eyes of a patient met the criteria, the right eye was included.

### Assessment of optic disc hemorrhage

Each ODH was evaluated using ONH stereoscopic photographs taken during the study follow-up period by two glaucoma experts (K.R.S. and J.M.K.) independently who were blinded to each patient's clinical information and VF results. The no ODH group was defined as including eyes without ODH during the whole follow-up period. The single ODH group was defined as that with eyes in which ODH only occurred once, while the recurrent ODH group was defined as that with eyes with ≥ 1 ODHs within 1 year after the initial ODH. If eyes experienced multiple ODHs at the same time, but no recurrence was found later on, these eyes were included in the single ODH group.

### SS-OCTA examination

All subjects underwent SS-OCTA, which has an eye-tracking system, using the FastTrac motion correction software. It operates at a 1060-nm central wavelength and its scan speed is 100,000 A-scans per second, with an axial resolution of 6.3 µm and a transverse resolution of 20 µm in tissue to acquire volumetric scans. Its A-scan depth-penetration in tissue was 3.0 mm, and the machine included an enhanced-depth imaging function. Only qualified and reliable images were included, and those with a signal strength index of < 8, representing poor scan quality, motion artifacts, misalignment, or irregular optic disc boundaries, were excluded.

The methods of identifying parapapillary choroidal VD, intradisc VD, and whole-image VD (wiVD) have been previously introduced^15,16^. An 8-bit binary slab was generated based on the mean threshold algorithm of the ImageJ software (version 1.52; Wayne Rasband, National Institutes of Health, Bethesda, MD, USA), which automatically measured the threshold value as the average of the local grayscale distribution. After assigning white pixels to vessels and black pixels to the background, respectively, VDs were calculated as a percentage of vessel pixels within the ONH and β-parapapillary atrophy (β-PPA) area.

**Figure 1:**
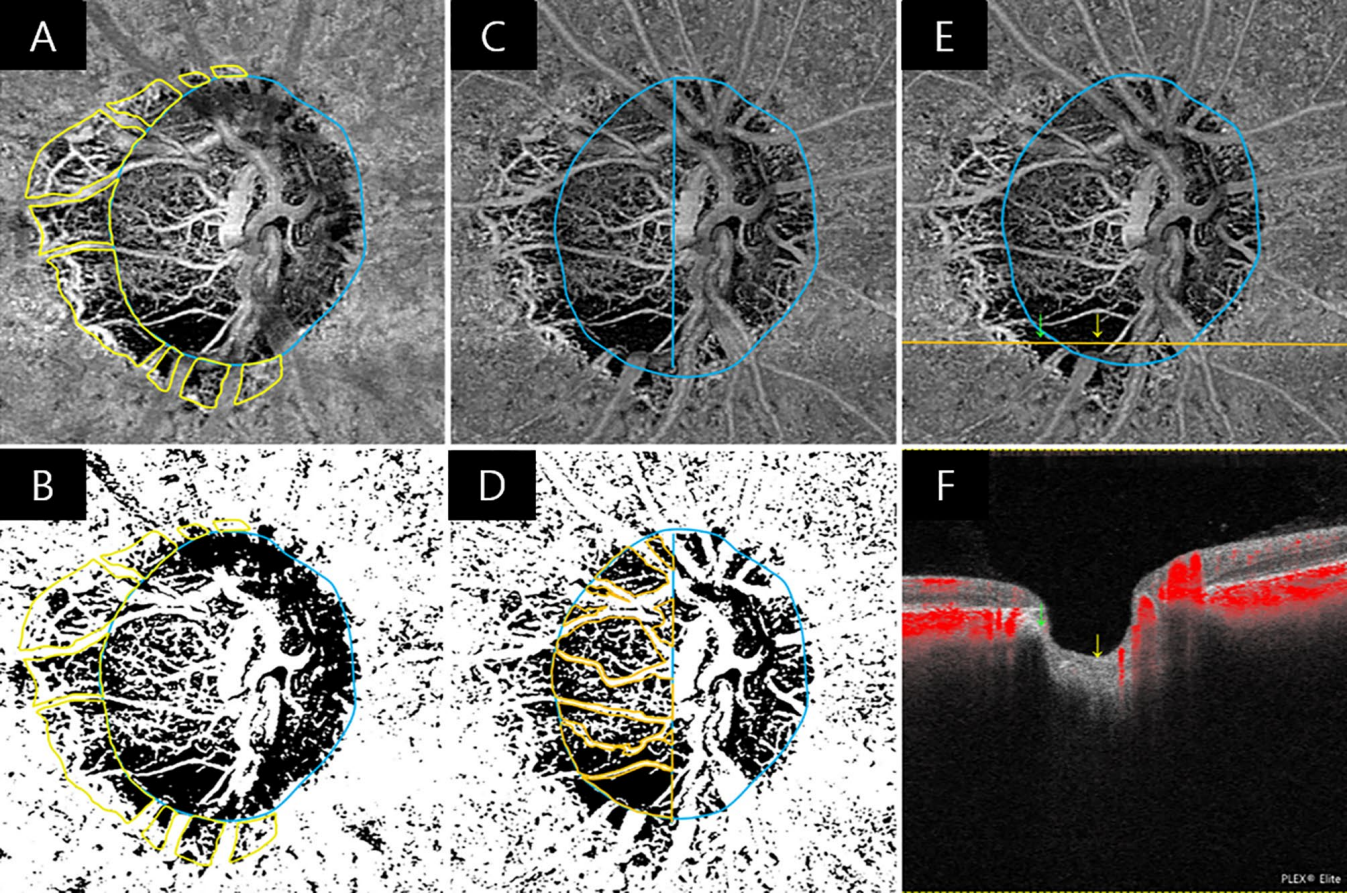
Measurements of parapapillary choroidal vessel density (VD) and intradisc VD and confirmation of the presence of choroidal microvascular dropout (CMvD), and optic disc microvascular dropout (MvD) in a primary open-angle glaucoma (POAG) eye with CMvD using swept-source optical coherence tomography angiography (SS-OCTA). (**A**) The blue ellipse line shows the optic nerve head (ONH) margin. The yellow outline indicates the β-parapapillary atrophy (β-PPA) margins, excluding large retinal vessels, which are applied to the SS-OCTA choroidal layer en-face image. The selected area of interest, excluding large retinal vessels, within the β-PPA was converted to a region of interest (ROI), (**B**) then applied to the 8-bit binary slab of an SS-OCTA choroidal layer en-face image. (**C**) The ROI within the ONH, excluding large retinal vessels, was manually demarcated on a whole-signal mode ONH en-face image. (**D**) These areas of interest were used to measure the intradisc VD (orange outline) using the ImageJ software. (**E**) The presence of CMvD (green arrow) and optic disc MvD (yellow arrow) was detected (**F**) using a horizontal B-scan that showed loss of microvasculature signal in the prelaminar tissue and anterior portion of lamina cribrosa.

Parapapillary choroidal VD was assessed using a 3.0 × 3.0 mm ONH choroidal layer en-face image automatically generated by layer segmentation of signals from the retinal pigment epithelium to the inner border of the sclera. To measure parapapillary choroidal VD within the β-PPA zone, the margins of the β-PPA zone were manually defined, while excluding the large projecting vessels from the overlying retinal layer within the β-PPA zone on en-face scanning laser ophthalmoscopy images using ImageJ software (Fig. 1A–B, yellow line)^15–17^.

Intradisc VD was evaluated using whole-signal mode ONH en-face images, which were automatically generated by signals below the inner limiting membrane. The boundary of the optic disc was manually delineated as the inner margin of the peripapillary scleral ring which was identified using scanning laser ophthalmology images, and then was applied to the same position of ONH en-face images using the ImageJ software. The temporal side of the vertical line passing across the optic disc center was used for the analysis because the deep intradisc microvasculature at the nasal side of the optic disc was difficult to visualize using OCTA due to large vessels and thick neuroretinal rim (Fig. 1C,D, orange line)^18–20^.

Average wiVD was assessed using a superficial slab of the ONH en-face image, extending from the inner limiting membrane to the posterior boundary of the inner plexiform layer. For the measurement of wiVD, the circumpapillary large vessels and optic disc margin were defined manually, then were excluded using ImageJ software.

The CMvD was defined as a focal sectoral capillary dropout with a circumferential width of > 200 µm or the width of the central retinal vein, with no visible microvascular network identified on the choroidal en-face images^7,21^. Optic disc MvD also was defined as a complete loss of the OCTA signal within the ONH, which is ≥ 200 µm in width and ≥ 100 µm in length^18^. For the evaluation of an optic disc MvD, visualization of the anterior lamina cribrosa (LC) was ensured in all horizontal B-scan images in order to verify the complete microvasculature loss throughout the deep layer of the ONH (Fig. 1E,F). Poor visibility of the LC was defined as < 70% visibility of the anterior LC surface within the Bruch's membrane opening^7,18^. If the anterior LC portion was poorly visualized at the area with complete OCTA signal loss in the horizontal B-scan images, the eyes were excluded from further analyses^18^. Two independent observers (J.Y.L. and J.W.S.), who were blinded to the clinical, VF, and RNFL details of the patients, identified MvDs. Disagreements between the observers were resolved by a third adjudicator (K.R.S.).

### Statistical analysis

Baseline characteristics are expressed using mean ± standard deviation values. The demographics and clinical characteristics were compared among the no ODH, single ODH, and recurrent ODH groups. For continuous variables, one-way analysis of variance with post-hoc tests (Tukey’s or Dunnett T3 methods) was used for the comparison of subgroups, as appropriate. For categorical data, chi-squared tests were performed. The kappa (κ) coefficient was calculated to evaluate inter-observer agreement in determining CMvD and intradisc MvD, respectively. Odds ratios (ORs) were calculated for the association between putative clinical factors, including OCTA-driven parameters and ODH using logistic regression analysis. Variables with *P* < 0.10 by univariate analyses were included in the multivariate models. To develop the final multivariate model, a backward elimination process was used and adjusted ORs with 95% confidence intervals (CIs) were calculated.

## Results

A total of 301 eyes were initially included in the study. Thirty-five eyes (11.6%) with poor-quality OCTA images were excluded because the ONH margin and intradisc VD quantification could not be clearly depicted by the software. Finally, 59 POAG eyes with single ODH, 40 POAG eyes with recurrent ODH, and 167 POAG eyes without ODH during the mean follow-up period of 5.4 years were included. Inter-examiner agreements regarding the determination of the presence of CMvD and intradisc MvD were excellent (*k* = 0.910, *P* < 0.001; *k* = 0.904, *P* < 0.001, respectively).

The demographics and baseline clinical characteristics of subjects were compared among the no ODH, single ODH, and recurrent ODH groups in Table 1. There were no significant differences among the three groups in age, sex, central corneal thickness, axial length, baseline IOP, and mean and peak IOPs during follow-up. However, no ODH group showed lower baseline VF mean deviation (MD) and thinner baseline RNFL thickness compared to the ODH group (all *P* < 0.05). The time between the detection of ODH in photographs and OCTA imaging was 2.2 ± 2.4 years and 3.4 ± 2.3 years in the single and recurrent ODH groups, respectively, with no significant difference between the groups (*P* = 0.288).

**Table 1:** Baseline characteristics of patients with primary open-angle glaucoma with and without optic disc hemorrhage (ODH).

	Group A: no ODH (n = 167)	Group B: single ODH (n = 59)	Group C: recurrent ODH (n = 40)	*P* value	Post-Hoc Test		
					*P*_*A-B*_	*P*_*A-C*_	*P*_*B-C*_
Age	58.01 ± 13.01	61.32 ± 10.19	58.33 ± 11.69	0.259			
Male	108 (64.7)	29 (49.2)	24 (60.0)	0.118			
Central corneal thickness	542.92 ± 44.53	540.31 ± 48.13	542.83 ± 29.78	0.946*			
Axial length	25.21 ± 1.74	24.97 ± 1.58	24.98 ± 1.63	0.788			
Baseline IOP	15.46 ± 4.10	14.98 ± 3.12	15.85 ± 5.48	0.554*			
Mean IOP	14.01 ± 2.27	14.92 ± 2.46	14.04 ± 1.93	0.304			
Peak IOP	17.86 ± 3.78	19.76 ± 4.18	19.87 ± 3.23	0.231			
Follow-up period	5.39 ± 2.60	5.52 ± 2.43	6.35 ± 1.96	0.116*			
Family history of glaucoma	19 (11.4)	7 (11.9)	5 (12.5)	0.989			
Hypertension	41 (24.6)	17 (28.8)	10 (25.0)	0.934			
Diabetes mellitus	29 (17.4)	16 (27.1)	7 (17.5)	0.264			
Cerebrovascular accident	2 (1.2)	3 (5.1)	0 (0.0)	0.123			
Ischemic heart disease	2 (1.2)	2 (3.4)	0 (0.0)	0.370			
Migraine	6 (3.6)	2 (3.4)	1 (2.6)	0.931			
Cold extremity	1 (0.6)	0 (0.0)	0 (0.0)	0.732			
Anticoagulant	15 (9.0)	11 (18.6)	2 (5.1)	0.071			
VF MD	− 7.43 ± 7.65	− 4.54 ± 4.89	− 3.32 ± 3.86	0.001*	**0.021**	**0.002**	0.619
Global RNFLT	72.54 ± 13.73	76.85 ± 10.43	83.13 ± 9.56	< 0.001*	0.068	** < 0.001**	**0.009**
Superior	86.29 ± 21.23	94.02 ± 17.49	103.89 ± 18.17	** < 0.001**	**0.039**	** < 0.001**	**0.045**
Inferior	80.12 ± 22.73	87.03 ± 20.95	90.98 ± 17.57	**0.013**	0.141	**0.023**	0.654

*IOP* intraocular pressure, *VF* visual field, *MD* mean deviation, *RNFLT* retinal nerve fiber layer thickness.

Comparison was performed using one-way analysis of variance with post-hoc tests (Tukey or *Dunnett T3).

Statistically significant values are in bold.

At the time of OCTA imaging, the three groups (no ODH, single ODH, and recurrent ODH) showed similar degrees of glaucomatous damage, without a significant difference in VF MD or RNFL thickness (Table 2). The proportions of CMvD, optic disc MvD, and β-PPA were not different among them. Further, parapapillary choroidal VD and wiVD did not differ among the 3 groups. However, eyes with ODH had significantly lower intradisc VDs than those without ODH (37.29 ± 6.01% vs. 39.49 ± 6.11%; *P* = 0.021) despite their similar degrees of glaucoma severity. The single and recurrent ODH groups did not show a significant difference in intradisc VD.

**Table 2:** Comparison of optical coherence tomography (OCT), visual field (VF), swept-source OCT angiography (SS-OCTA) parameters among POAG eyes with or without optic disc hemorrhage (ODH) at the time of SS-OCTA imaging.

	Group A: no ODH (n = 167)	Group B: single ODH (n = 59)	Group C: recurrent ODH (n = 40)	*P* value	*Post-Hoc Test*		
					*P*_*A-B*_	*P*_*A-C*_	*P*_*B-C*_
VF MD	− 7.86 ± 7.22	− 6.79 ± 5.63	− 7.21 ± 6.27	0.555	0.579	0.923	0.981
Global RNFLT	69.17 ± 14.25	71.46 ± 10.35	72.82 ± 8.63	0.989	0.888	0.756	0.930
Presence of PPA	162 (97.0)	59 (100.0)	38 (95.0)	0.271			
Presence of CMvD	92 (55.1)	37 (62.7)	27 (67.5)	0.277			
Presence of optic disc MvD	42 (25.1)	16 (27.1)	10 (25.0)	0.987			
Intradisc VD	39.49 ± 6.11	37.19 ± 6.16	37.44 ± 5.47	**0.021**	**0.038**	**0.040**	0.977
Parapaillary choroidal VD	46.35 ± 6.77	45.34 ± 6.59	45.76 ± 5.83	0.638	0.627	0.302	0.950
Whole image VD	40.38 ± 6.42	41.76 ± 6.30	41.64 ± 6.20	0.262	0.326	0.508	0.995

*VF* visual field, *MD* mean deviation, *RNFLT* retinal nerve fiber layer thickness, *PPA* parapapillary atrophy, *CMvD* choroidal microvascular dropout, *MvD* microvascular dropout, *VD* vessel density.

Comparison was performed using one-way analysis of variance with post-hoc tests (Tukey or *Dunnett T3).

Statistically significant values are in bold.

Table 3 presents clinical variables associated with the occurrence of ODH using univariate and multivariate analyses. In the univariate analysis, earlier glaucoma stages represented by functional and structural parameters (VF MD [OR, 1.096; 95% CI, 1.043–1.151; *P* < 0.001] and global RNFL thickness [OR, 1.045; 95% CI, 1.023–1.068; *P* < 0.001]) and lower intradisc VD (OR, 0.939; 95% CI, 0.900–0.980; *P* = 0.004) showed associations with ODH occurrence. After multivariate analysis, a better VF MD at baseline (OR, 1.150; 95% CI, 1.055–1.254; *P* = 0.002) and lower intradisc VD (OR, 0.863; 95% CI, 0.812–0.918; *P* < 0.001) were independently associated with ODH occurrence.

**Table 3:** Univariate and multivariate logistic regression analyses for clinical factors associated with occurrence of optic disc hemorrhage during follow-up in eyes with primary open-angle glaucoma.

	Univariate analysis	Multivariate analysis		
	Odds ratio (95% CI)	*P value*	Odds ratio (95% CI)	*P* value
Age	1.014 (0.993–1.035)	0.197		
Male	0.629 (0.379–1.045)	0.073		
Central corneal thickness	0.999 (0.993–1.005)	0.627		
Axial length	0.910 (0.767–1.080)	0.282		
Baseline IOP	0.988 (0.929–1.050)	0.691		
Mean IOP	0.919 (0.834–1.013)	0.088		
Baseline VF MD	1.096 (1.043–1.151)	** < 0.001**	1.150 (1.055–1.254)	**0.002**
Baseline global RNFLT	1.045 (1.023–1.068)	** < 0.001**		
Presence of PPA	1.497 (0.285–7.865)	0.634		
Presence of CMvD	1.491 (0.893–2.489)	0.127		
Presence of optic disc MvD	0.997 (0.564–1.765)	0.993		
Intradisc VD	0.939 (0.900–0.980)	**0.004**	0.863 (0.812–0.918)	** < 0.001**
Parapaillary choroidal VD	0.976 (0.940–1.013)	0.204		
Whole image VD	1.035 (0.993–1.078)	0.103		

*CI* confidence interval, *IOP* intraocular pressure, *VF* visual field, *MD* mean deviation, *RNFLT* retinal nerve fiber layer thickness, *PPA* parapapillary atrophy, *CMvD* choroidal microvascular dropout, *MvD* microvascular dropout, *VD* vessel density.

Significant values are in bold.

We analyzed clinical factors associated with the frequency of ODH during follow-up using logistic regression analyses (Table 4). There was no difference in the VD of ONH evaluated by SS-OCTA between eyes with single and recurrent ODH. The frequency of ODHs during follow-up was only significantly associated with the baseline earlier stage of structural change in POAG eyes (global RNFL thickness, OR, 1.066; 95% CI, 1.019–1.116; *P* = 0.006).

**Table 4:** Univariate logistic regression analysis for clinical factors associated with recurrence of optic disc hemorrhage during follow-up in eyes with primary open-angle glaucoma.

	Univariate analysis	
	Odds ratio (95% CI)	*P value*
Age	0.976 (0.936–1.013)	0.202
Male	0.672 (0.297–1.522)	0.341
CCT	1.002 (0.992–1.013)	0.677
Axial length	1.020 (0.753–1.381)	0.901
Baseline IOP	1.054 (0.954–1.164)	0.299
Mean IOP	1.020 (0.852–1.220)	0.830
Baseline VF MD	1.067 (0.965–1.181)	0.207
Baseline global RNFLT	1.066 (1.019–1.116)	**0.006**
Presence of PPA	1.523 (0.438–2.614)	0.954
Presence of CMvD	1.105 (0.471–2.593)	0.818
Presence of optic disc MvD	0.927 (0.369–2.325)	0.871
Intradisc VD	1.007 (0.940–1.079)	0.838
Parapaillary choroidal VD	1.017 (0.954–1.084)	0.610
Whole image VD	0.997 (0.934–1.064)	0.922

*CI* confidence interval, *CCT* central corneal thickness, *IOP* intraocular pressure, *VF* visual field, *MD* mean deviation, *RNFLT* retinal nerve fiber layer thickness, *PPA* parapapillary atrophy, *CMvD* choroidal microvascular dropout, *MvD* microvascular dropout, *VD* vessel density.

Significant values are in bold.

**Figure 2:**
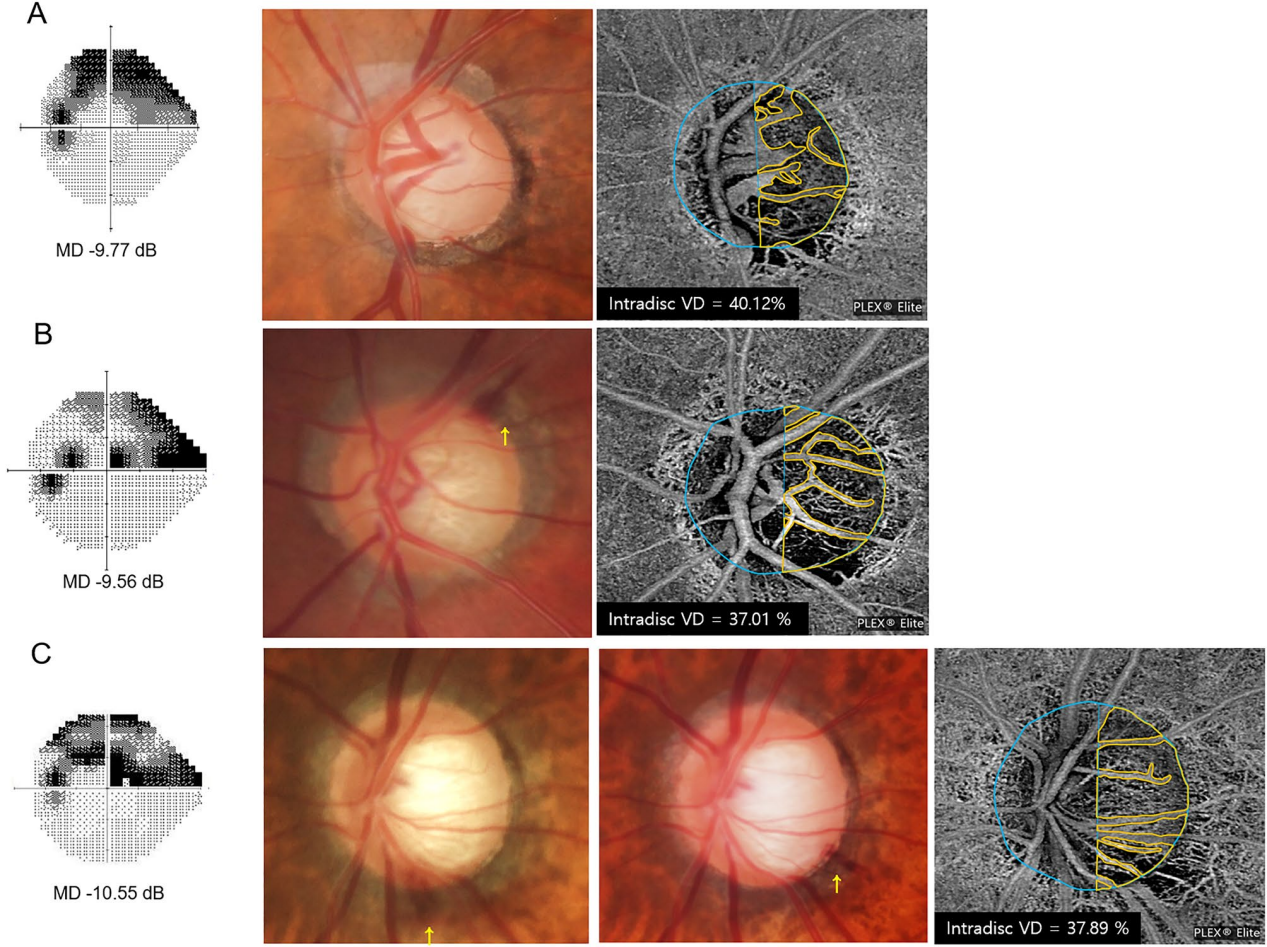
Representative primary open-angle glaucoma eyes with and without optic disc hemorrhage (ODH)**.** (**A**) A 65-year-old female patient without ODH during follow-up in her left eye. (**B**) A 64-year-old male patient with a single ODH in his left eye. (**C**) A 66-year-old male patient with recurrent ODH during follow-up in his left eye. All these eyes presented with choroidal microvascular dropout (CMvD) in the inferotemporal area and superior visual field (VF) loss with similar degrees of damage represented by VF mean deviation (MD). On an optical coherence tomography angiography (OCTA) whole-signal mode en-face image of the optic nerve head (ONH), the blue ellipse indicates the ONH margin and the blue vertical line denotes boundaries of vessel density (VD) measurement areas within the ONH passing across the optic disc center. The boundaries of the ONH (orange outline), excluding large retinal vessels, were manually demarcated. These areas of interest were used to measure the intradisc VD. The intradisc VDs were 37.01% and 37.89% in eyes with single ODH and recurrent ODH, respectively, which were lower than that in an eye without ODH (40.12%).

Figure 2 presents reduced intradisc VD in eyes with ODH compared to those without ODH. A left eye of a 65-year-old female patient with POAG without ODH during follow-up showed a superior hemifield scotoma with a VF MD of − 9.19 dB at baseline and an intradisc VD of 40.12%. In comparison, a 64-year-old male patient with OAG in his left eye and single ODH during follow-up and a 66-year-old male patient with OAG in his left eye and recurrent ODH during follow-up, matched for age and baseline VF severity, presented with relatively lower intradisc VDs (37.01% and 37.89%, respectively).

## Discussion

We evaluated the association between ODH and intradisc VD in POAG eyes. On SS-OCTA scans, the intradisc VD measurements were significantly lower in the ODH group than the no ODH group, whereas the parapapillary choroidal VD and wiVD values were similar between the two groups. Moreover, the occurrence of ODH was significantly associated with a lower intradisc VD based in multivariate analyses. These findings suggest that eyes with ODH may have association with the microvasculature loss within the ONH region.

Despite the correlation between ODH and faster glaucoma progression^22–25^, the pathogenesis of ODH remains unclear. Recently, OCTA has been widely used to overcome the difficulty of exploring the ONH vasculature in glaucomatous eyes with ODH, and studies have reported decreased ONH-related vessel densities in them. Rao et al. reported that there was no difference in the whole en-face VD of ONH evaluated by spectral-domain OCTA between eyes with and without ODH in the early stage of glaucoma (VF MD = − 3.8 dB)^26^. The association between the presence of ODH history and reduced parapapillary VD in patients with normal-tension glaucoma was supported by Nitta et al.^27^ and Okamoto et al.^28^ Our results showed that the occurrence of ODH was related to lower intradisc VD in eyes with POAG. The underlying pathogenesis may not be fully elucidated in the present study, but we speculate the following scenario. Since ONH perfusion can be dependent on adjacent parapapillary choroidal circulation^29,30^, the presence of CMvD may indicate reduced ocular perfusion. Also, the strong spatial correlation between CMvD and optic disc MvD has been reported by previous studies^18,31^. Such data may partly explain the ODH group having a higher rate of patients with CMvD and lower intradisc VD.

Our study found that parapapillary choroidal VD and wiVD measurements did not differ between eyes with and without ODH. As the parapapillary choroid is closely related to ONH perfusion, insufficient blood flow within the parapapillary choroid can result in decreased blood flow to the ONH and glaucomatous damage^32^. In addition, diminished blood flow to the parapapillary choroid (i.e., CMvD) was associated with a great degree of glaucomatous damage^33^. Because eyes with and without ODH had similar glaucoma severities at the time of SS-OCTA imaging, the parapapillary choroidal VD might not have shown a difference between the two groups. However, this requires careful interpretation due to the transient nature of ODH because ODH may have occurred and been absorbed before or in the middle of CMvD and optic disc MvD development during follow-up.

In the current study, recurrent ODH occurred in 40.4% (40/99) of ODH eyes during 5.4 years. As ODH is known to precede glaucomatous changes^34^, recurrent ODH may be associated with more extensive glaucomatous changes. Ishida et al.^35^ reported that their recurrent ODH group showed more progressive VF changes than the single ODH group after a mean follow-up period of 5.6 years. We speculated that optic disc perfusion, as represented by the intradisc VD, might be more decreased in the recurrent ODH group. However, we were unable to demonstrate any significant difference between the single ODH and recurrent ODH groups with regard to intradisc VD, parapapillary choroidal VD, and wiVD. This may be due to the different mechanism among various types of ODH or due to the similar degree of glaucoma severity at the time of SS-OCTA imaging. Further studies are needed to clarify speculations about the mechanism and clinical significance of recurrent ODH in POAG patients.

Kurvinen et al. evaluated peripapillary retinal blood flow at the time of ODH detection and 6 months later using scanning laser Doppler flowmetry^36^. They found that reduced flow at the time of ODH and increased flow after resorption supported the vascular etiology of ODH. Park et al. compared eyes with ODH at the border of localized RNFL defects and those with ODH not related to localized RNFL defects using disc fluorescein angiography^37^ and found prolonged arteriovenous transit time and vessel filling defects or delayed filling in eyes with ODH accompanying RNFL defects, implying blood flow stasis at the disc margin proximal to where ODH occurred. In our study, whether reduced intradisc VD is the result or cause of ODH is unclear; we only acquired SS-OCTA images during follow-up after the technology was introduced in 2020. Nonetheless, despite OCTA’s inherent limitations in accurately representing the nature of vascular changes, we speculate that capillary occlusion and resultant hemorrhage may have led to the reduction of intradisc VD or blood flow, and subsequent stasis could have caused ODH. To evaluate the temporal relationship between parapapillary choroidal VD/intradisc VD/RNFLT changes and ODH detection, prospective studies with baseline SS-OCTA imaging are required.

Our finding is in agreement with previous studies^38,39^ in which the frequency of ODH increased from an early to moderate stage (i.e., higher VF MD value at baseline) and decreased with progression toward an advanced stage of glaucoma in eyes in which ODH was no longer detected, consistent with prior research that found that ODH was detected more frequently in early to moderate glaucoma than advanced glaucoma^39,40^.

It is possible that ODH and lower intradisc VD may be a marker for higher susceptibility to glaucoma progression, but their association with further neural damage remains unclear. In fact, a previous study did not reveal a difference in the incidence of hemorrhages between treated and untreated groups over time^41^. A recent report showed that localized VF defects were found to occur prior to ODH and to continue after the event^22^. In the present study, the ODH group showed less advanced disease stage at baseline compared to non-ODH group. However, at the time of OCTA imaging after similar duration of follow-up period, the ODH group showed a similar degree of disease severity. This observation suggests that ODH and/or reduced intradisc VD may be both a result of progressive glaucomatous damage and an indicator of possible subsequent progression.

The following limitations should be considered when interpreting our results. First, patients assigned to the no ODH group could have had ODH in the past when they were not monitored or in the period between study visits. Therefore, the number of ODHs may have been underestimated in some patients. To reduce this, we included only patients who had been regularly followed up for ≥ 3 years with optic disc photographs taken every 6 months. Second, factors associated with hypertension (e.g., blood pressure and use of antihypertensive medication) could have affected ODH or VD measurements. Although no difference in the proportion of patients with hypertension was found between the groups with and without ODH, further studies that include hypertension data are warranted. Third, the OCTA algorithm includes large vessels along the capillaries within ONH in the estimation of VD. To minimize the potential effects of artifacts, such as large retinal vessels, neuroretinal rim shadowing, and projections, only images with well-visualized optic disc margins and anterior LC in the horizontal B-scans that detected complete loss of a microvascular structure were included. Our results may not be generalizable to settings that use different OCT/OCTA devices; however, we used SS-OCTA as it is able to clearly visualize the intradisc microvasculature with deeper penetration and higher resolution than other approaches^42,43^. Lastly, the β-PPA area has been associated not only with the severity of glaucoma but also with the visibility and density of parapapillary choroidal vessels^15,16^. Although areal measurements were not conducted in this study, we speculated that estimation of parapapillary choroidal VD better reflected ischemic insults within the β-PPA than did β-PPA area itself. Additionally, the density of parapapillary choroidal vessels and the presence of CMvD were not significantly different between the groups with and without ODH.

In conclusion, POAG eyes with ODH showed reduced intradisc VD compared to those without ODH when measured by SS-OCTA. Eyes with POAG in an earlier disease stage or those with lower intradisc VDs should be monitored for the possibility of ODH occurrence.

## Data Availability

The datasets used and/or analyzed during the current study are available from the corresponding author upon request.
